# The legs of the young heart stool: how motor competence, muscle strength, and physical activity support cardiorespiratory fitness in preschoolers?

**DOI:** 10.3389/fspor.2026.1829678

**Published:** 2026-05-14

**Authors:** Weizhen Gao, Cindy Hui-Ping Sit, Xu Wen, Xinyu Wang, Cong Huang, Peige Song, Jane Jie Yu

**Affiliations:** 1 Department of Sports Science, College of Education, Zhejiang University, Hangzhou, China; 2 Department of Sports Science and Physical Education, Faculty of Education, The Chinese University of Hong Kong, Hong Kong SAR, China; 3 School of Public Health, Zhejiang University School of Medicine, Hangzhou, China; 4 Centre for Global Health, Usher Institute, University of Edinburgh, Edinburgh, United Kingdom

**Keywords:** accelerometry, cardiorespiratory fitness, motor competence, muscle strength, preschoolers

## Abstract

**Introduction:**

Cardiorespiratory fitness (CRF) is a key marker of early childhood health, yet its determinants are poorly understood. This study aimed to identify the multi-dimensional impacts of physical activity (PA), motor competence, and muscle strength on CRF in preschoolers, considering age and sex differences.

**Methods:**

A total of 329 preschoolers (aged 3.0–6.9 years) were initially recruited in China between April and June in 2024, with a final sample of 300 children included in the analysis. CRF, PA levels (light, moderate, and vigorous), and motor competence components (manual dexterity, aiming and catching, and balance) were assessed with the PREFIT 20 m shuttle run, the ActiGraph GT3X+ accelerometer (for seven consecutive days), and the Movement Assessment Battery for Children-Second edition (MABC-2), respectively. Upper- and lower- limb strength was evaluated by grip dynamometer and standing long jump test, respectively. Generalized linear mixed models and hierarchical regressions were employed to analyze the data, stratified by age and sex.

**Results:**

Hierarchical models showed that demographics explained 32.3% of CRF variance, motor competence 7.6%, muscle strength 3.0% and PA added 2.3%. Specifically, manual dexterity, balance, handgrip strength, standing long jump, and VPA were identified as significant independent predictors. Stratified analyses revealed that manual dexterity and VPA were the primary predictors for boys and girls, respectively, with muscle strength emerging as an additional key determinant for both sexes in the older cohort.

**Discussion:**

CRF in preschoolers is influenced by an interplay of factors, with VPA, motor competence, and muscle strength being key. The pattern of predictors varies by age and sex, highlighting the need for tailored interventions to promote CRF effectively.

## 1 Introduction

The preschool years are a critical period for establishing lifelong health trajectories, with cardiorespiratory fitness (CRF) recognized as a foundational component of physical health (1, 2). Longitudinal studies provide compelling evidence that lower levels of CRF in youth are associated with a greater risk of adverse cardiometabolic outcomes in adulthood, underscoring the importance of this early developmental window (3–5). Despite its significance, most research on CRF correlates has focused on school-aged children, leaving a relative lack of high-quality evidence for preschoolers (6). Existing studies in this younger population have often used broad physical fitness indices, with few undertaking a comprehensive investigation of the specific factors that influence CRF (7).

Theoretical frameworks, such as the developmental model of motor competence by Stodden, posit a synergistic interplay between motor competence, physical activity (PA), and health-related physical fitness (8). While originally conceptualized with a lifespan trajectory often applied to school-aged youth, the preschool years act as a critical early window within this model, where foundational motor skills first begin to either constrain or facilitate sustained PA engagement and subsequent fitness adaptations (9). Within this model, CRF is viewed not in isolation, but as a physiological outcome driven by specific behavioral and functional precursors (10, 11). Biologically, PA serves as the primary stimulus for cardiorespiratory adaptation (12). However, a child's capacity to engage in sustained PA is fundamentally constrained by their movement proficiency and muscle strength. Motor competence provides the necessary motor skills to execute movements efficiently, while muscle strength provides the power and structural capacity to sustain these activities (9, 13). Without adequate motor competence and muscle strength, children may struggle to achieve the exercise intensity required to improve CRF. Consequently, a comprehensive understanding of early-life CRF requires examining how these three interrelated components-PA, motor competence, and muscle strength—collectively contribute to CRF.

The relationships within this developmental system are also unlikely to be uniform across all children. The preschool years are characterized by rapid physiological maturation, suggesting that the primary determinants of CRF may differ as children age (14, 15). Furthermore, well-documented sex-based differences in motor performance and activity patterns emerge during early childhood, which may contribute to distinct developmental pathways for boys and girls (16). An aggregated analysis that overlooks these fundamental demographic factors might therefore mask important, group-specific patterns.

Therefore, this study aimed to investigate the distinct and joint contributions of PA, motor competence, and muscle strength to CRF in preschoolers. Specifically, we sought to: (1) determine the relative importance of these dimensions and identify which specific outcomes serve as the critical drivers of CRF; and (2) examine whether these relationships are moderated by age and sex. We hypothesized that higher level of PA and motor competence, and greater muscle strength will be positively associated with better CRF. Additionally, we expect the strength of these associations to vary by age and sex. By elucidating these age- and sex- specific pathways, our findings will provide a refined empirical foundation to understand the development of CRF among preschoolers and to aid the design of tailored effective intervention programs for improving CRF in early childhood.

## 2 Materials and methods

### 2.1 Participants

This study enrolled 329 children aged 3–6 years from four kindergartens in Zhejiang Province, China, selected through purposeful sampling. Data collection was conducted between April and June 2024. To minimize disruption to the regular kindergarten curriculum and prevent physical and cognitive fatigue in the young participants, a flexible testing schedule was adopted rather than a rigid sequential protocol. The assessments were integrated into the children's daily free-play time over a period of 1–2 weeks for each class. Parents signed written informed consents for children's participation. Parent-reported demographic data, including children's birth and sex, were collected. Inclusion criteria comprised: (1) preschool enrollment in target institutions, (2) parental/guardian provision of written informed consent, and (3) completion of baseline health screening confirming absence of exclusion conditions. Exclusion criteria comprised: (1) diagnosed disabilities affecting motor function (e.g., autism spectrum disorder), and (2) acute health conditions that could interfere with assessment participation (e.g., recent injuries). Data from 29 children were missing or incomplete due to the occurrence of various circumstances (absence, sick or injured, refusal to participate, etc.) during data collection. Eventually, complete data from 300 (91.2%) out of the 329 children and their families initially recruited for participation were included in the final data analyses in this study. The sample size was shown to have sufficient power (>0.80) to detect moderate associations (*f*^2^ = 0.15) in hierarchical regression models, given an intraclass correlation coefficient of 0.05 and allowing for a 10% attrition rate.

### 2.2 Outcomes and measurements

CRF was assessed as the dependent variable, while PA, motor competence, and muscle strength were measured as independent predictors. For data quality control, all assessments were administered by a team of trained graduate students specializing in pediatric exercise science. Prior to data collection, examiners completed a comprehensive 2-day training workshop led by the principal investigator.

Additionally, an online survey was administered using the SoJump (a platform available for online research in China) available for online research to collect information regarding the children's characteristics. The data collection process was designed to ensure that all relevant information was obtained while respecting participants' privacy and consent.

#### 2.2.1 Cardiorespiratory fitness

CRF was measured using the PREFIT 20 m shuttle run test. The PREFIT protocol has demonstrated high test-retest reliability and excellent feasibility specifically in preschool children aged 3–6 years. This protocol is currently widely accepted as a feasible field-based measure for estimating CRF in early childhood (17, 18). During the test, participants ran back and forth at an initial speed of 6.5 km/h on two tracks 20 m apart, and then in increments of 0.5 km/h per minute. During the test, participants ran back and forth according to the timing of a beep from the compact disc recorder. The test was conducted once. The 20 m shuttle run is considered more fun than running around a track, and it accurately reflects the CRF of younger children. Because we were working with younger children, the track was marked, and a research assistant helped lead the children so they could master the pace of running. The outcome measure was the total number of laps completed (with a higher number of laps indicating superior CRF), which was recorded for the final analysis.

#### 2.2.2 Physical activity

To assess PA, we used the ActiGraph GT3X+ accelerometer to collect data continuously over 7 days. The device was worn on the children's right hip and was only removed during bathing or sleeping. The accelerometer was configured with the following parameters: a sampling frequency of 30 Hz and a 15 s epoch; non-wear time was defined according to the Choi algorithm; valid data required at least 480 min of wear time per day; and a minimum of 4 days (3 weekdays and 1 weekend day) of valid data were included in the analysis. PA intensities were classified using triaxial accelerometer cutpoints adapted from Pate et al.: low physical activity (LPA) defined as 200–419 counts/15 s, Moderate physical activity (MPA) as 420–841 counts/15 s, and VPA as ≥842 counts/15 s (19). Although a child's exercise capacity matures rapidly between ages 3 and 6, these established cut-points are widely used because they effectively capture age-appropriate relative intensities for the preschool population as a whole (19). Parents and teachers received both verbal and written instructions on proper accelerometer usage. Data initialization and analysis were performed using ActiLife software (version 6.13.3). The final PA outcomes were presented as the average actual time (in minutes) spent in LPA, MPA, and VPA on all valid days.

#### 2.2.3 Motor competence

The Movement Assessment Battery for Children-Second Edition (MABC-2), developed by Henderson et al., is an internationally standardized assessment tool for evaluating motor competence in children aged 3–14 years (20). The MABC-2 demonstrates excellent test–retest reliability (ICC = 0.92) and content validity (I-CVI = 0.985) in Chinese children aged 3–6 years (21). In this study, the MABC-2 assessment focused on children in the 3–6-year age range, evaluating motor competence through three dimensions including manual dexterity, aiming and catching, and balance. The manual dexterity dimension included three tasks (i.e., coin placement, bead threading, and drawing); the aiming and catching dimension included two tasks (i.e., beanbag throwing and beanbag grasping); and the balance dimension consisted of three tasks (i.e., walking on tiptoes, one-leg balancing, and carpet hopping). The final motor competence outcomes included the percentile scores for the overall motor competence and each of the three dimensions (Manual Dexterity, Aiming & Catching, and Balance). These percentile scores were derived from the standard scores, with higher percentiles indicating better motor competence (21).

#### 2.2.4 Muscle strength

Muscle strength was assessed using outcomes widely recognized in international health consensus, specifically upper limb strength and lower limb strength (22). Upper limb strength was evaluated through grip dynamometer. During the test, children were instructed to stand with their feet naturally apart and arms hanging down, hold the grip strength dynamometer, and exert maximum force to tightly grip the upper and lower handles. Given that young children often struggle to gauge their hand strength accurately, each child underwent two trials with each hand, with the highest value recorded as the final grip strength score. Lower limb strength was assessed using the standing long jump test. Children were required to stand behind the take-off line with their feet naturally apart, perform a half-squat, and then swing their arms to jump forward forcefully. The distance from the take-off line to the landing position of the child's heels was measured as the test score. After two consecutive trials, the maximum distance was recorded as the final standing long jump score. These two outcomes are also utilized in the physical fitness assessment for young children in China and have demonstrated good reliability and validity (23).

#### 2.2.5 Demographic and health variables

Child demographic and health information was collected via an online questionnaire. Parents reported each child's date of birth, sex, birth weight, birth length, and gestational age. The current height and weight of preschool children were measured in kindergartens. Body mass index (BMI) was calculated from current height and weight (kg/m^2^) and converted to a standardized score on a 100-point scale based on norm standard (23).

### 2.3 Data analyses

Descriptive statistics, including means, standard deviations, and percentages, were calculated as appropriate. Continuous variables are presented as mean and standard deviation, while categorical variables are presented as frequency (percentage). Independent samples *t*-tests were used to compare sex differences in all outcome measures. Partial Pearson correlation analyses were used to explore the linear relationships among CRF, PA, motor competence, and muscle strength of preschoolers after controlling for sex and kindergarten. A generalized linear mixed model (GLMM) was constructed with CRF as the dependent variable. Fixed effects comprised: (1) child demographics, (2) PA, (3) motor competence, and (4) muscle strength. The inclusion of kindergarten as a random effect addressed potential cluster effects arising from shared institutional environments. This modeling approach allowed simultaneous estimation of individual predictors while controlling for between-kindergarten variability. Following GLMM analysis, hierarchical linear regression models were employed with variables entered sequentially: child demographics first, followed by PA, motor competence, and finally, muscle strength. This stepwise approach quantified the contributions (Δ*R*^2^) and standardized regression coefficients (*β* values) across different dimensions. Additional stratified analyses were conducted separately by age groups (3.0–4.9 years vs. 5.0–6.9 years) to explore potential developmental shifts in CRF predictors, as physiological maturation and motor competence acquisition progress is fast during this period. Within these age groups, we further examined the impact of sex on the predictors of CRF to observe how the influence of various predictors on CRF might differ across different age and sex subgroups. All statistical analyses were performed using SPSS 26.0, with the significance level set at a two-sided *p* < 0.05.

## 3 Ethics statement

Utilizing a cross-sectional design that complied with the principles of the Helsinki Declaration as revised in 2024, this study was approved by the Research Ethics Committee of the Department of Psychology and Behavioral Sciences at the University (Approval No. 2022/056).

## 4 Results

### 4.1 Demographic and health-related characteristics of the participants

As shown in Table 1, this study included 300 preschoolers aged 3–6 years (152 males, 50.7%; 148 females, 49.3%). Neonatal parameters averaged 3.23 ± 0.50 kg for birth weight, 50.50 ± 3.86 cm for length, and 38.81 ± 1.87 weeks for gestational age, with no significant group differences (all *p* > 0.05). Sex-specific analyses revealed significant differences: males exhibited greater handgrip strength (*p* < 0.001), higher MPA (*p* < 0.001), along with more VPA (*p* < 0.05), while females demonstrated superior manual dexterity (*p* < 0.05) and balance (*p* < 0.001). There were no significant sex differences in the rest of outcomes (all *p* > 0.05).

**Table 1:** Demographic characteristics and health-related outcomes in preschoolers by Sex (mean ± SD).

Characteristics	Total (*N* = 300)	Boys (*N* = 152)	Girls (*N* = 148)	*t*-value
Demographics				
Age (years)	5.16 ± 0.77	5.18 ± 0.80	5.14 ± 0.75	0.391
BMI (kg/m^2^)	15.40 ± 1.73	15.55 ± 1.69	15.24 ± 1.75	1.542
Birth weight (kg)	3.23 ± 0.50	3.26 ± 0.49	3.19 ± 0.51	1.236
Birth length (cm)	50.50 ± 3.86	50.71 ± 4.26	50.28 ± 3.39	0.936
Gestational age (weeks)	38.81 ± 1.87	38.71 ± 1.79	38.92 ± 1.96	−0.960
Cardiorespiratory fitness				
20 m shuttle run (laps)	16.57 ± 9.33	16.51 ± 9.97	16.64 ± 8.69	−0.117
Physical activity				
Light PA (min/d)	76.07 ± 14.90	77.42 ± 14.25	74.68 ± 15.46	1.593
Moderate PA (min/d)	52.80 ± 14.52	55.21 ± 14.23	50.33 ± 14.43	2.948**
Vigorous PA (min/d)	19.31 ± 10.58	20.63 ± 10.39	17.96 ± 10.64	2.201*
Motor Competence				
Manual Dexterity (0-100)	49.73 ± 26.33	46.22 ± 24.80	53.35 ± 27.45	-2.358**
Aiming and Catching (0-100)	41.55 ± 27.69	43.30 ± 28.69	39.73 ± 26.59	1.112
Balance (0-100)	52.09 ± 23.82	47.05 ± 22.73	57.30 ± 23.89	−3.801**
Muscle strength				
Handgrip Strength (kg)	7.08 ± 2.11	7.44 ± 2.30	6.71 ± 1.84	3.030**
Standing Long Jump (cm)	91.74 ± 18.68	93.33 ± 20.02	90.11 ± 17.11	1.488

* *p* < 0.05.

** *p* < 0.01.

### 4.2 Associations of CRF with other outcomes

**Figure 1:**
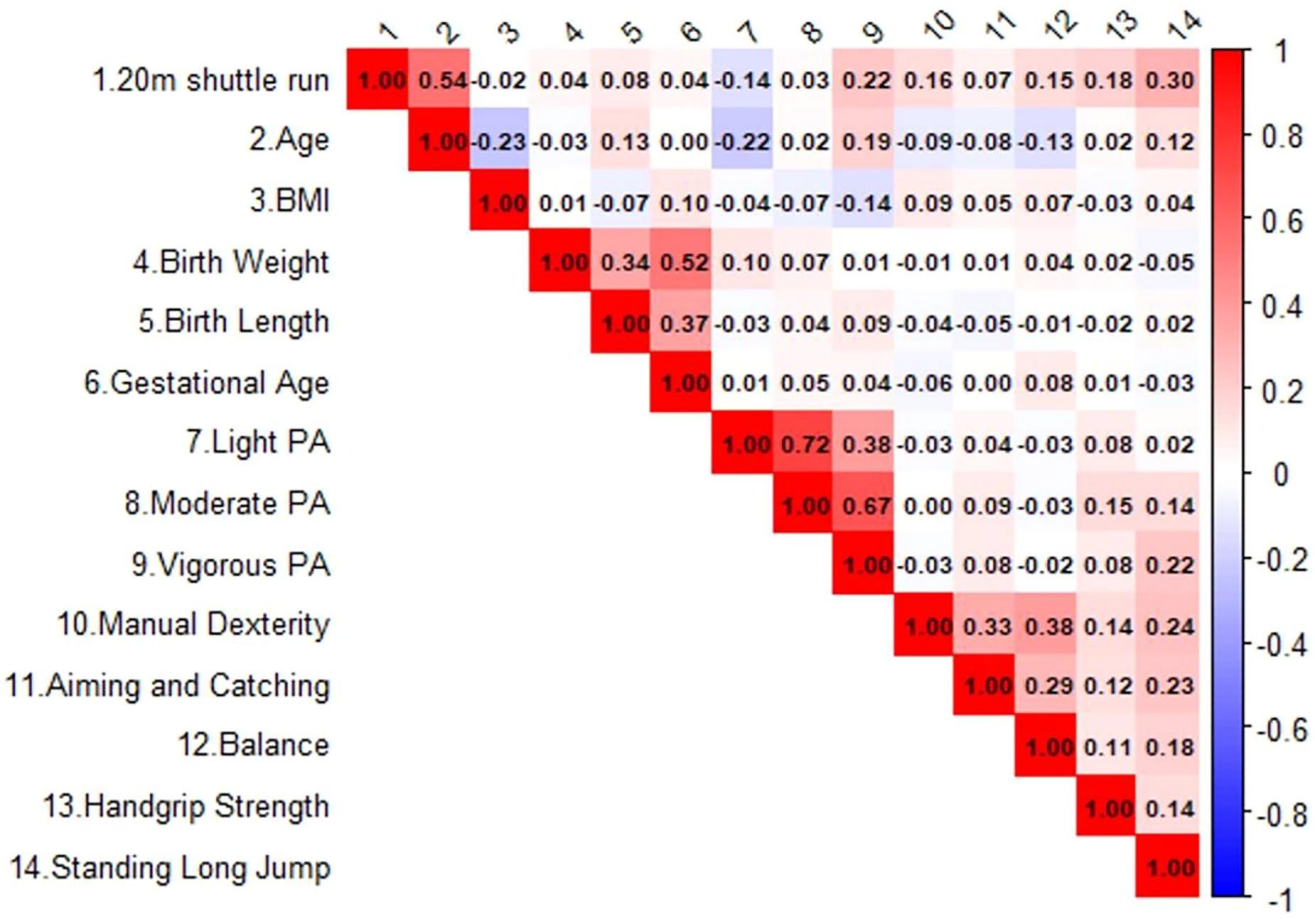
Correlation matrix of key variables in preschoolers.

After controlling for sex and kindergarten, partial correlation analysis showed CRF was most strongly associated with age (*r* = 0.536, *p* < 0.001). Other significant positive correlates included standing long jump (*r* = 0.297, *p* < 0.001), VPA (*r* = 0.220, *p* < 0.001), manual dexterity (*r* = 0.160, *p* = 0.037), and handgrip strength (*r* = 0.145, *p* = 0.021) (Figure 1).

Preliminary analyses using aggregate measures indicated that overall motor competence was a significant predictor of CRF (*β* = 1.268, *p* < 0.05). To identify the precise contributors within each domain, we further stratified the analyses by specific sub-dimensions (Table 2). The GLMM results confirmed these findings, with age (*β* =0.568), VPA (*β* =0.192), handgrip strength (*β* =0.146), standing long jump (*β* =0.136), manual dexterity (*β* = 0.113), and balance (*β* = 0.114) all emerging as significant and independent predictors of CRF (all *p* < 0.05).

**Table 2:** Factors associated with cardiorespiratory fitness in preschoolers using generalized mixed linear models.

Fixed effect	Estimate(*β*)	Standard error	*t*-value	95% confidence interval	
				Lower	Upper
Demographics					
Sex = boy	0.002	0.102	0.018	−0.198	0.202
Sex = girl	0 (ref.)				
Age	0.568**	0.056	10.172	0.458	0.677
BMI scores	0.103*	0.051	2.017	0.002	0.204
Birth weight	0.058	0.060	0.978	−0.059	0.175
Birth length	−0.018	0.053	−0.344	−0.123	0.086
Gestational age	0.006	0.060	0.098	−0.111	0.123
Physical activity					
Light PA	−0.010	0.076	−0.133	−0.160	0.140
Moderate PA	−0.110	0.091	−1.212	−0.290	0.069
Vigorous PA	0.192**	0.069	2.795	0.057	0.327
Motor competence					
Manual Dexterity	0.113*	0.056	2.032	0.003	0.222
Aiming and Catching	−0.044	0.054	−0.810	−0.149	0.062
Balance	0.114*	0.058	1.974	0.000	0.228
Muscle strength					
Handgrip Strength	0.146**	0.052	2.808	0.043	0.248
Standing Long Jump	0.136**	0.051	2.682	0.036	0.236

Categorical variables: boy = 1, girl = 0 (reference); The dependent variable in this model is cardiorespiratory fitness, measured by the 20 m shuttle run test.

* *p* < 0.05.

** *p* < 0.01.

### 4.3 Hierarchical predictors of CRF in preschoolers

Hierarchical regression analysis quantified the unique contribution of each domain (Table 3). The demographic model (age, sex, BMI) explained 32.3% of the variance in CRF. The subsequent inclusion of predictor domains significantly improved the model, with motor competence contributing the largest unique variance (Δ*R*^2^ = 7.6%), followed by muscle strength (Δ*R*^2^ = 3.0%) and physical activity (Δ*R*^2^ = 2.3%). In the final model, age remained the strongest predictor, but VPA, manual dexterity, balance, handgrip strength, and standing long jump were all significant independent contributors. When the sample was stratified by age, a clear developmental shift in CRF predictors emerged (Table 4). In the younger cohort (3.0–4.9 years, *n* = 124), VPA (*β* = 0.199, *p* = 0.008) and manual dexterity (*β* = 0.196, *p* = 0.020) were the only significant predictors in the final model (*R*^2^ = 21.3%). In contrast, for the older cohort (5.0–6.9 years, *n* = 176), the predictive pattern shifted, with standing long jump (*β* = 0.238, *p* = 0.002) and handgrip strength ((*β* = 0.168, *p* = 0.013) emerging as the dominant predictors, and the final model explaining a larger portion of variance (*R*^2^ = 26.3%).

**Table 3:** Predictors of cardiorespiratory fitness in preschoolers using hierarchical linear regression models.

Predictors	Model 1 *β*	Model 2 *β*	Model 3 *β*	Model 4 *β*
Demographics				
Age	0.587**	0.568**	0.608**	0.596**
BMI scores	0.154*	0.161*	0.137*	0.128*
Physical activity				
VPA		0.114*	0.122**	0.087*
Motor competence				
Manual Dexterity			0.141**	0.108*
Balance			0.149**	0.123*
Muscle strength				
Handgrip Strength				0.118*
Standing Long Jump				0.124*
*R* ^2^	0.323	0.346	0.422	0.452
Δ*R*^2^		0.023	0.076	0.030
F change	69.229**	5.424*	13.196**	6.927**

The dependent variable in this model is cardiorespiratory fitness, measured by the 20 m shuttle run test. The models are built in a hierarchical manner, with each subsequent model adding new predictors to the previous model. Model 1 includes demographic variables, Model 2 adds PA, Model 3 adds motor competence, and Model 4 adds muscle strength.

* *p* < 0.05.

** *p* < 0.01.

**Table 4:** Predictors of cardiorespiratory fitness in preschoolers using hierarchical linear regression models by Age groups.

	3.0–4.9 Years (*N* = 124)				5.0–6.9 Years (*N* = 176)			
Predictors	Model 1 *β*	Model 2 *β*	Model 3 *β*	Model 4 *β*	Model 1 *β*	Model 2 *β*	Model 3 *β*	Model 4 *β*
Demographics								
BMI scores	0.159	0.178	0.140	0.119	0.092	0.099	0.076	0.068
Physical activity								
VPA		0.201**	0.228**	0.199**		0.107	0.105	0.041
Motor competence								
Manual Dexterity			0.225*	0.196*			0.193*	0.134
Balance			0.117	0.094			0.132	0.104
Muscle strength								
Handgrip Strength				0.008				0.168*
Standing Long Jump				0.129				0.238**
*R* ^2^	0.025	0.085	0.178	0.213	0.009	0.030	0.131	0.263
Δ*R*^2^		0.060	0.103	0.024		0.021	0.101	0.132
F change	2.991	6.927**	7.524**	1.936	1.484	3.011	8.770**	9.832**

The dependent variable in this model is cardiorespiratory fitness, measured by the 20 m shuttle run test. The models are built in a hierarchical manner, with each subsequent model adding new predictors to the previous model. Model 1 includes demographic variables, Model 2 adds physical activity, Model 3 adds motor competence, and Model 4 adds muscle strength.

* *p* < 0.05.

** *p* < 0.01.

Further stratification by sex within each age group revealed additional nuances. For boys aged 3.0–4.9 years, manual dexterity was the primary predictor (*β* = 0.275, *p* = 0.032), whereas for girls of the same age, CRF was driven by VPA (*β* = 0.342, *p* = 0.023). In the older group (5.0–6.9 years), boys' CRF was most strongly related to leg power and grip strength (with dexterity also contributing), while girls' CRF was significantly predicted by grip strength (*β* = 0.249, *p* = 0.011) and VPA (*β* = 0.372, *p* = 0.001). All models maintained acceptable multicollinearity thresholds (VIF=1.03–1.48).

## 5 Discussion

This study of 300 Chinese preschoolers (age 3–6) identified several key determinants of early CRF. In general, our results confirmed that motor competence, muscle strength, and PA were all significant independent predictors of CRF after controlling for demographic factors as we expected. Notably, based on the variance explained in our hierarchical models, motor competence emerged as the strongest contributor, followed by muscle strength and PA. While confirming the importance of motor competence, muscle strength, and PA, our primary contribution is the novel evidence that the relative influence of these factors shifts significantly with age. Specifically, we found that VPA and motor competence are paramount for CRF in younger preschoolers (3.0–4.9 years), whereas muscle strength emerges as the dominant predictor in their older counterparts (5.0–6.9 years). These findings underline that early CRF is jointly shaped by developmental stage, motor competence, muscle strength, and PA.

The results underscore the pivotal role of motor competence. Overall, children with higher overall motor competence demonstrated superior CRF levels. Among the specific dimensions, manual dexterity and balance exhibited the strongest associations with CRF. The emergence of manual dexterity as a significant predictor in the younger cohort (3.0–4.9 years) warrants further investigation regarding the relationship between motor competence and CRF in early childhood. While the neurocognitive link between fine motor skills and aerobic fitness has been documented in school-aged children, evidence in preschoolers remains limited (24). We speculate that manual dexterity may act as an early developmental marker for the neural maturity required for efficient movement. In school-aged children, it is typically proficient gross motor skills (e.g., object control) that facilitate participation in PA and subsequent fitness gains (8, 25). However, our data indicate that preschoolers are in a distinct developmental stage characterized by rapid neural maturation but often immature gross motor coordination. As noted in recent studies, Chinese preschoolers often demonstrate superior manual dexterity but underdeveloped object control skills compared to Western norms (26, 27). Consequently, manual dexterity may capture variance in neuromotor development that underdeveloped ball skills cannot yet detect. These findings indicate that the specific motor contributors to CRF evolve with age, and that future longitudinal research should examine to identify exactly when gross motor skills begin to supersede manual dexterity as the primary predictor.

Beyond motor competence, muscle strength emerged as a robust correlate. In our full-sample models, both handgrip strength and standing long jump distance predicted CRF. Notably, these strength measures became the dominant predictors in 5.0–6.9-year-olds, whereas they were negligible in 3.0–4.9-year-olds. This developmental shift makes sense: as children grow, their muscle mass and power increase, allowing CRF to play a larger role. Stronger leg muscles, for example, help sustain shuttle-run efforts more efficiently. Our findings echo previous research linking muscle to CRF. For instance, Wittekind et al. showed that skeletal muscle mass predicts peak VO₂ in youth (28), and Reisberg et al. (2024) reported that handgrip strength correlates with shuttle-run performance in preschoolers (29). Children with higher grip strength typically possess greater overall muscular capacity, which facilitates better fatigue resistance during the progressive stages of the shuttle run. Practically, activities that build strength and power, such as climbing, jumping, or simple resistance play, may help older preschoolers boost both muscle strength and CRF.

VPA was another independent contributor to preschool CRF, while LPA and MPA did not demonstrate a similarly strong relationship. The World Health Organization recommends that children aged 3–5 years engage in at least 180 min of PA per day, including a minimum of 60 min of MVPA, with an emphasis on energetic play (30). Our finding, that only the vigorous component of MVPA was significantly related to CRF, suggests that MPA (e.g., walking or casual play) may not consistently elevate heart rate and oxygen uptake enough to drive improvements in CRF at this age. This pattern aligns with longitudinal data indicating that only VPA (e.g., running games or active tag) translate into measurable fitness gains (31, 32). Another study has shown that both MPA and VPA significantly influence preschoolers' CRF (33). Therefore, we suggest that practice should prioritize opportunities for VPA, such as organized running drills, chase games, or obstacle courses, to maximize early CRF.

The effect of age on CRF is consistent with developmental physiology. As children grow, increases in heart size, lung capacity, and lean body mass raise maximal oxygen uptake (34). Across the early lifespan, CRF shows a robust, age-related improvement: in preschoolers, shuttle-run performance increased linearly with age as demonstrated by the PREFIT norms in Spain (14). Armstrong and Welsman report that, peak VO₂ rises steeply from late childhood through adolescence, which also reflected these maturation processes (34). Furthermore, an important insight was the age-specific pattern of predictors in our study. Among the younger preschoolers (3.0–4.9 years), VPA and motor competence (especially manual dexterity) were the key drivers of CRF, whereas in 5.0–6.9-year-olds, muscle strength was more influential. There is little direct prior research on such fine-grained age differences, but our findings have parallels in the literature. Leppänen et al. reported that for 4–5-year-olds, higher VPA predicted short-term fitness gains, in line with our observation that VPA dominated in the younger cohort (31). Similarly, developmental models suggest that younger children rely heavily on motor competence to engage in activity (35); as those skills mature, older children's higher fitness levels become increasingly constrained by muscle development (14). Our findings provide critical evidence for designing age-specific public health guidelines. For children aged 3.0–4.9, interventions should prioritize creating opportunities for vigorous, skill-based play to simultaneously build motor foundations and cardiorespiratory health. Conversely, for children aged 5.0–6.9, public health initiatives and preschool curricula should strategically incorporate muscle-strengthening activities (e.g., climbing, structured jumping games), as this appears to be the most potent lever for improving CRF in this pre-school-entry age group, thereby better preparing them for a healthy life trajectory.

Although average CRF did not differ by sex in our cohort, we uncovered sex-specific differences in predictor patterns. Even within each age band, boys and girls appeared to benefit from different emphases. In 3.0–4.9-year-olds, boys' CRF was primarily associated with manual dexterity, while girls' CRF was driven by VPA. Among 5.0–6.9-year-olds, boys' CRF was tied to leg power and grip strength (with dexterity contributing), whereas girls' CRF was significantly related to both strength and sustained VPA. These patterns echo prior observations: Latorre-Roman et al. also found minimal sex differences in preschoolers' mean CRF but noted that boys tend to have higher strength and activity while girls excel in balance and manual dexterity (16). Moreover, Reisberg et al. observed that in preschoolers, higher VPA predicted later fitness more strongly in girls than in boys (29). Taken together, our results suggest that even at preschool age, a single intervention may inadvertently favor one sex over the other. To ensure all children benefit, public health programs must be nuanced. Educators and parents might therefore consider sex-sensitive approaches, for example, emphasizing agility and coordination drills for young boys and actively promoting vigorous, high-energy play for young girls (16). Ignoring these differences may perpetuate or even widen early-life health disparities.

To the best of our knowledge, this is the first empirical study to examine the joint contributions of PA, motor competence, and muscle strength to CRF in preschoolers from a multi-dimensional perspective. We assessed CRF and a wide range of potential predictors at a personal level using standardized and validated field tests (e.g., 20-m shuttle run, MABC-2) and analyzed the data with rigorous mixed and hierarchical models. Few studies have simultaneously examined PA, motor competence, muscular strength, and demographics in preschoolers. Nonetheless, certain limitations apply. The cross-sectional design means we cannot infer causality; for example, while VPA predicts CRF, it is also possible that fitter children naturally enjoy more intense play. We did not directly measure family, community, or school environments, which could influence CRF and mediate the observed associations. For instance, factors such as parental activity behaviors, home environment, community resources, and preschool curricula could play significant roles. While we included many relevant variables, other factors (e.g., nutrition, genetic background, preschool curriculum) were not examined and could influence CRF. Future research should include longitudinal tracking to confirm these developmental trajectories and randomized controlled trials to test the efficacy of age- and sex-tailored interventions. Additionally, investigating the role of the wider socio-ecological environment (e.g., preschool curriculum, built environment, parental behaviors) is a critical next step for creating comprehensive public health solutions.

In summary, preschoolers' CRF is determined by a constellation of interrelated factors including motor competence (manual dexterity and balance), muscle strength (both upper and lower limb strength), and PA (VPA). The associations of CRF with the abovementioned factors vary as a child grows and by sex. By aligning interventions with children's age- and sex-specific abilities and needs, for example, focusing on vigorous, skill-based play in the youngest children and incorporating strength-building activities in the oldest, healthy CRF trajectories in preschoolers can be more effectively fostered.

## Data Availability

The raw data supporting the conclusions of this article will be made available by the authors, without undue reservation.

## References

[1] Raghuveer G Hartz J Lubans DR Takken T Wiltz JL Mietus-Snyder M Cardiorespiratory fitness in youth: an important marker of health: a scientific statement from the American Heart Association. Circulation. (2020) 142(7):e101–18. 10.1161/CIR.0000000000000866 32686505 PMC7524041

[2] Schmutz EA Leeger-Aschmann CS Kakebeeke TH Zysset AE Messerli-Bürgy N Stülb K Motor competence and physical activity in early childhood: stability and relationship. Front Public Health. (2020) 8:39. 10.3389/fpubh.2020.00039 32154207 PMC7047434

[3] Gao Z Wang R. Children’s motor skill competence, physical activity, fitness, and health promotion. J Sport Health Sci. (2019) 8(2):95–7. 10.1016/j.jshs.2018.12.002 30997254 PMC6451052

[4] Myers J Cadenas-Sanchez C Ross R Kokkinos P. The critical role of cardiorespiratory fitness in disease prevention. J Sports Med Phys Fitness. (2024) 64(12):1361–71. 10.23736/s0022-4707.24.16159-2 39287581

[5] Ortega FB Labayen I Ruiz JR Kurvinen E Loit HM Harro J Improvements in fitness reduce the risk of becoming overweight across puberty. Med Sci Sports Exerc. (2011) 43(10):1891–7. 10.1249/MSS.0b013e3182190d71 21407124

[6] Jiang T Zhao G Fu J Sun S Chen R Chen D Relationship between physical literacy and cardiorespiratory fitness in children and adolescents: a systematic review and meta-analysis. Sports Med. (2025) 55(2):473–85. 10.1007/s40279-024-02129-7 39579330 PMC11947022

[7] Lv W Fu J Zhao G He Z Sun S Huang T A cohort study of factors influencing the physical fitness of preschool children: a decision tree analysis. Front Public Health. (2023) 11:1184756. 10.3389/fpubh.2023.1184756 38074715 PMC10701283

[8] Stodden DF Goodway JD Langendorfer SJ Roberton MA Rudisill ME Garcia C A developmental perspective on the role of motor skill competence in physical activity: an emergent relationship. Quest. (2008) 60(2):290–306. 10.1080/00336297.2008.10483582

[9] Robinson LE Stodden DF Barnett LM Lopes VP Logan SW Rodrigues LP Motor competence and its effect on positive developmental trajectories of health. Sports Med. (2015) 45:1273–84. 10.1007/s40279-015-0351-6 26201678

[10] Bardid F Utesch T Lenoir M. Dynamics between motor competence, cardiorespiratory fitness and weight status in children: a cross-lagged longitudinal analysis. J Sport Exerc Psychol. (2018) 40(S1):S19. 10.1123/jsep.2018-0169

[11] Cattuzzo MT dos Santos Henrique R Ré AHN de Oliveira IS Melo BM de Sousa Moura M Motor competence and health related physical fitness in youth: a systematic review. J Sci Med Sport. (2016) 19(2):123–9. 10.1016/j.jsams.2014.12.004 25554655

[12] Gralla MH McDonald SM Breneman C Beets MW Moore JB. Associations of objectively measured vigorous physical activity with body composition, cardiorespiratory fitness, and cardiometabolic health in youth: a review. Am J Lifestyle Med. (2019) 13(1):61–97. 10.1177/1559827615624417 30627080 PMC6311603

[13] Souilla L Avesani M Boisson A Requirand A Matecki S Vincenti M Cardiorespiratory fitness, muscle fitness, and physical activity in children with long QT syndrome: a prospective controlled study. Front Cardiovasc Med. (2023) 9:1081106. 10.3389/fcvm.2022.1081106 36712265 PMC9874118

[14] Cadenas-Sanchez C Intemann T Labayen I Peinado AB Vidal-Conti J Sanchis-Moysi J Physical fitness reference standards for preschool children: the PREFIT project. J Sci Med Sport. (2019) 22(4):430–7. 10.1016/j.jsams.2018.09.227 30316738

[15] Jurov I Demšar J. Factors affecting maximal oxygen uptake in prepubertal children: a systematic review and meta-analysis. BMC Pediatr. (2024) 24(1):550. 10.1186/s12887-024-05013-5 39192196 PMC11348524

[16] Latorre Roman PA Moreno Del Castillo R Lucena Zurita M Salas Sanchez J Garcia-Pinillos F Mora Lopez D. Physical fitness in preschool children: association with sex, age and weight status. Child Care Health Dev. (2017) 43(2):267–73. 10.1111/cch.12404 27666424

[17] Cadenas-Sanchez C Martinez-Tellez B Sanchez-Delgado G Mora-Gonzalez J Castro-Piñero J Löf M Assessing physical fitness in preschool children: feasibility, reliability and practical recommendations for the PREFIT battery. J Sci Med Sport. (2016) 19(11):910–5. 10.1016/j.jsams.2016.02.003 26947061

[18] Mora-Gonzalez J Cadenas-Sanchez C Martinez-Tellez B Sanchez-Delgado G Ruiz JR Léger L Estimating VO2max in children aged 5–6 years through the preschool-adapted 20-m shuttle-run test (PREFIT). Eur J Appl Physiol. (2017) 117(11):2295–307. 10.1007/s00421-017-3717-7 28932901

[19] Pate RR Almeida MJ McIver KL Pfeiffer KA Dowda M. Validation and calibration of an accelerometer in preschool children. Obesity. (2006) 14(11):2000–6. 10.1038/oby.2006.234 17135617

[20] Henderson S Sugden D Barnett A. Movement Assessment Battery for Children-2 s Edition (Movement ABC-2). London, UK: The Psychological Corporation (2007).

[21] Hua J Wu Z Gu G Meng W. Applied research on a complete set of assessment tools for children’s motor coordination ability. Chin J Epidemiol. (2012) 33(10):1010–5. 10.3760/cma.j.issn.0254-6450.2012.10.004

[22] Luz C Cordovil R Rodrigues LP Gao Z Goodway JD Sacko RS Motor competence and health-related fitness in children: a cross-cultural comparison between Portugal and the United States. J Sport Health Sci. (2019) 8(2):130–6. 10.1016/j.jshs.2019.01.005 30997259 PMC6450916

[23] CISS. National physical fitness testing standards (Revised in 2023) (in Chinese). Available online at: https://www.ciss.cn/tzgg/info/2023/32672.html (Accessed August 10, 2023).

[24] Klupp S Grob A Mohring W. Aerobic fitness and fine motor skills are related to switching and updating in typically developing children. Psychol Res. (2023) 87(5):1401–16. 10.1007/s00426-022-01749-w 36264512 PMC10227109

[25] Barnett LM Lai SK Veldman SL Hardy LL Cliff DP Morgan PJ Correlates of gross motor competence in children and adolescents: a systematic review and meta-analysis. Sports Med. (2016) 46(11):1663–88. 10.1007/s40279-016-0495-z 26894274 PMC5055571

[26] Ke L Du W Wang Y Duan W Hua J Barnett AL. The movement ABC-2 test in China: comparison with UK norms for 3–10 year olds. Res Dev Disabil. (2020) 105:103742. 10.1016/j.ridd.2020.103742 32711248

[27] Zheng Y Ye W Korivi M Liu Y Hong F. Gender differences in fundamental motor skills proficiency in children aged 3–6 years: a systematic review and meta-analysis. Int J Environ Res Public Health. (2022) 19(14):8318. 10.3390/ijerph19148318 35886186 PMC9324170

[28] Wittekind SG Powell AW Opotowsky AR Mays WW Knecht SK Rivin G Skeletal muscle mass is linked to cardiorespiratory fitness in youth. Med Sci Sports Exerc. (2020) 52(12):2574–80. 10.1249/MSS.0000000000002424 32520872

[29] Reisberg K Riso EM Animagi L Jurimae J. Longitudinal associations between physical activity and sedentary time and cardiorespiratory and muscular fitness in preschoolers. J Funct Morphol Kinesiol. (2024) 9(4):199. 10.3390/jfmk9040199 39449493 PMC11503317

[30] WHO. WHO guidelines on physical activity and sedentary behaviour. Available online at: https://www.who.int/publications/i/item/9789240015128 (Accessed November 25, 2020). 33369898

[31] Leppänen MH Henriksson P Delisle Nystrom C Henriksson H Ortega FB Pomeroy J Longitudinal physical activity, body composition, and physical fitness in preschoolers. Med Sci Sports Exerc. (2017) 49(10):2078–85. 10.1249/MSS.0000000000001313 28538260

[32] Burgi F Meyer U Granacher U Schindler C Marques-Vidal P Kriemler S Relationship of physical activity with motor skills, aerobic fitness and body fat in preschool children: a cross-sectional and longitudinal study (ballabeina). Int J Obes (Lond). (2011) 35(7):937–44. 10.1038/ijo.2011.54 21448128

[33] Riso EM Toplaan L Viira P Vaiksaar S Jurimae J. Physical fitness and physical activity of 6–7-year-old children according to weight status and sports participation. PLoS One. (2019) 14(6):e0218901. 10.1371/journal.pone.0218901 31237932 PMC6592557

[34] Armstrong N Welsman JO. Traditional and new perspectives on youth cardiorespiratory fitness. Med Sci Sports Exerc. (2020) 52(12):2563–73. 10.1249/MSS.0000000000002418 32735109 PMC7664977

[35] Zeng N. Relationships among Physical Activity, Motor Skill Competence, Cardiovascular Fitness, Perceived Competence, and Cognition in Preschool Children. Minneapolis, MN: University of Minnesota (2018).

